# COVID-19: A Cross-Sectional Study of Healthcare Students’ Perceptions of Life during the Pandemic in the United States and Brazil

**DOI:** 10.3390/ijerph18179217

**Published:** 2021-09-01

**Authors:** Laura A. Geer, Rachel Radigan, Guilherme de Lima Bruneli, Lucas Sampaio Leite, Rosalie Barreto Belian

**Affiliations:** 1 School of Public Health, SUNY Downstate Health Sciences University, Brooklyn, NY 11203, USA; rachel.radigan@downstate.edu; 2 Keizo Asami Immunopathology Laboratory, Federal University of Pernambuco, Recife 50670-901, Brazil; guilherme.bruneli@ufpe.br (G.d.L.B.); lucas.sampaio@ufpe.br (L.S.L.); rosalie.belian@ufpe.br (R.B.B.)

**Keywords:** COVID-19 risk perceptions, healthcare students’ perceptions, health risk factors, infection prevention, health promotion, international collaboration, intercultural learning

## Abstract

Societal influences, such as beliefs and behaviors, and their increasing complexity add to the challenges of interactivity promoted by globalization. This study was developed during a virtual global educational exchange experience and designed for research and educational purposes to assess personal social and cultural risk factors for students’ COVID-19 personal prevention behavior and perceptions about life during the pandemic, and to inform future educational efforts in intercultural learning for healthcare students. We designed and implemented a cross-sectional anonymous online survey intended to assess social and cultural risk factors for COVID-19 personal prevention behavior and students’ perceptions about life during the pandemic in public health and healthcare students in two public universities (United States n = 53; Brazil n = 55). Statistically significant differences existed between the United States and Brazil students in degree type, employment, risk behavior, personal prevention procedures, sanitization perceptions, and views of governmental policies. Cultural and social differences, risk messaging, and lifestyle factors may contribute to disparities in perceptions and behaviors of students around the novel infectious disease, with implications for future global infectious disease control.

## 1. Introduction

Developing human resources capable of contributing positively to solving global public health problems is a current challenge including the evolution of education of healthcare students’ needs with the shifting global climate created by the pandemic [1,2]. In this sense, higher education courses for healthcare students need to be deeply transformed to comprehend and deal with worldwide problems, regarding local, regional, and global contexts [1,3]. Moreover, societal and cultural influences add to the challenges of interactivity promoted by globalization [4].

The declaration of the Coronavirus Disease 2019 (COVID-19) pandemic from the World Health Organization (WHO) brought about physical and social distancing and quarantine regulations in efforts to slow the transmission of the disease [5,6]. Individuals were thus forced to decrease their interactions with close family and friends. With “stay-at-home” orders in place, anxiety and loneliness complicated daily life [7]. Psychological impacts were seen in the general population throughout the world. University students are one of the most vulnerable populations for economic, social, and mental health determinants from the COVID-19 pandemic [1]. High rates of stress, anxiety, and depression were witnessed in university populations. Insecurities around social life, academic success, career and employment possibilities fuel these vulnerabilities [1].

Consequences of the pandemic were also seen in the economy with rising unemployment rates resulting in hardships for many people. “Fear, misinformation, and a general sense of individual and social loss of control” was felt across the nation as a result of the spread of COVID-19 [7]. Specifically in students, fear of mental health impacts routed in coping, anxiety, mood, and guilt, were major barriers of adherence to testing, social distancing, and self-isolation. According to the Abraham Maslow hierarchy, one must meet one’s basic physiological, safety, and love and belonging needs before achieving higher functions such as academic success [8]. COVID-19 is a source of tension on students’ development and success [3].

The recent coronavirus pandemic is a prime example of geographic and cultural differences in risks for severe acute respiratory syndrome coronavirus 2 (SARS-CoV-2) transmission and COVID-19 impacts including socio-economic factors, the influence of political regime, and underlying disease prevalence [9]. Such differences between countries warrant investigation of gaps in risk communication and messaging and a consideration of the real needs of populations to avoid misunderstandings and stigmatization [10]. In particular, universities must consider that students are not insulated from the economic and social disparities faced by majority populations in developing countries or recent immigrants, such as health inequities, health policies effects of poverty, and lack of sanitation [11]. Cross-cultural learning experiences promote the sharing of information, knowledge, and values, and, thus, contribute to preparing students as health professionals for collaborating in teams capable of solving global health problems. Herein, we present findings based on an international educational virtual exchange known as the State University of New York (SUNY) Collaborative Online International Learning (COIL) program, featuring a partnership between the Federal University of Pernambuco in Recife, Brazil, and the School of Public Health at SUNY Downstate Health Sciences University (DHSU) in Brooklyn, New York. The United States and Brazil were listed in the top twenty countries most affected by COVID-19. When looking at deaths per 100,000 population, Brazil was listed as the second-highest and the United States was the sixth-highest [12]. We extend the work based on a prior three-week-long international module that was carried out over two semesters in 2019 to include a survey of student perceptions about life during the pandemic [4].

This study was designed for research and educational purposes to compare the influence of cultural differences on COVID-19 prevention strategies among healthcare students. We consider this study a feasibility pilot to inform future educational efforts in intercultural learning for healthcare students.

## 2. Materials and Methods

### 2.1. Sample Characteristics

Our sample population was derived from healthcare students participating in COIL, consisting of a partnership between the Federal University of Pernambuco located in Recife in northeast Brazil and the SUNY DHSU School of Public Health located in Brooklyn, New York in the United States [4].

### 2.2. Survey Development and Implementation

**Figure 1:**
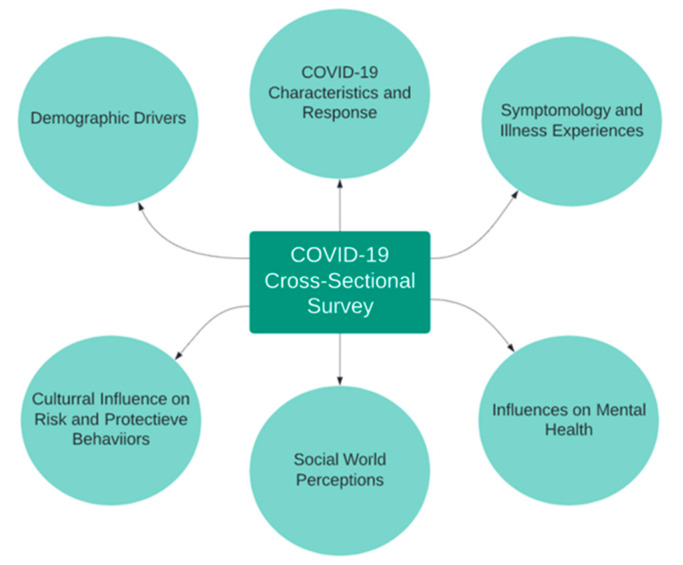
Cross-sectional survey flow of student perceptions examined through self-report.

The ad hoc survey was developed across both countries to address self-reported perceptions of student life during the pandemic. Themes assessed are consistent with those from a recent COVID-19 survey in youth around social and behavioral influences [13]. In our survey, participants were asked 40 questions, which collated around the following topics: (1) demographics (e.g., age, employment), (2) COVID-19 physical characteristics and response, e.g., test availability, (3) symptomatology and illness experiences including number and type of symptoms, (4) cultural influence on risk and protective behaviors such as sanitization procedures, personal protective equipment (PPE) use, (5) social world perceptions, i.e., social relationship activity, and (6) influences on mental health (Figure 1). Questions consisted of multiple-choice, multiple answers, ordinal scale, and open-ended.

In this cross-sectional study, we administered a cross-sectional survey to graduate-level public health and medical students from Brazil and the U.S. Students aged 18 years and older were emailed the survey, which was conducted online through Google Forms. Through this Google-operated forum, the survey was created using varying question types, and data were collected and organized. This short 20-min survey was fully anonymous and a regular part of an existing public health course at Downstate Health Sciences University. Students voluntarily answered this cross-sectional survey between October and November of 2020. At the end of the survey, participants were asked if they agreed to the use of their responses for research purposes.

### 2.3. Statistical Methods

Data were tabulated using Excel and analyzed using IBM SPSS Statistics Version 27 (IBM Corp, Armonk, NY, USA) Descriptive statistics were performed to determine sample characteristics and the distribution of variables used in the models. Bivariate analysis was performed to analyze factors comparing students from Brazil and the United States. For continuous variables, such as age, a t-test was used. For categorical data, an X^2^ test was used. All analyses were evaluated at the 0.05 alpha level.

## 3. Results

A total of 109 students responded, however, one was excluded due to not agreeing to have answers used as academic research. Of the valid responses, 53 (49%) were from students in the U.S. and 55 (51%) were from students in Brazil.

### 3.1. Demographics

The average age of all participants was 25.3 years (SD = 6.8) with 64% being female and 36% being male. Race and ethnicity were only recorded for the U.S. student population. The majority of U.S. students (85%) did not consider themselves to be Hispanic or Latino/a. All Brazilian students were born in Brazil. For U.S. students, 64% (n = 34) were born inside the U.S. with 36% (n = 19) being born outside of the US. Fourteen other countries were listed as a birthplace; some of the birth places listed were Bangladesh, Egypt, Guyana, and Haiti. Students from Brazil were more likely to be medical students (93%, n = 51), whereas those from the U.S. were more likely to be public health students (85%, n = 45). Employment was more common among the U.S. students versus Brazil before the pandemic (83% vs. 9%) as well as currently (67% vs. 11%), respectively. Among those employed in the U.S., 47% were satisfied with work while 53% were not satisfied. Among those employed in Brazil, 83% were satisfied with work and 17% were not satisfied. Among those unemployed in the U.S., 65% were seeking employment while 35% were not seeking employment. Among those unemployed in Brazil, 6% were seeking employment while 94% were not seeking employment. Additional demographic drivers can be seen in Table 1.

### 3.2. COVID-19 Physical Characteristics and Symptomatology

Students were asked questions relating to their experience with illness as a result of COVID-19, testing for COVID-19, and their opinions on policies (Table 2). COVID-19 symptomology did not differ significantly between students living in the U.S. versus Brazil. COVID-19 symptoms as demonstrated in the categorical grouping of symptoms in Table 2 elicit the students’ consequences of their direct experience with the disease. Fatigue and headache were among the most commonly reported symptoms, with chest pain and/or pressure being the least reported. For those that responded and took or were prescribed medication for COVID-19 treatment, qualitative results showed Tylenol or Ibuprofen were dominant medications among U.S. students, and Azithromycin or Ivermectin were dominant medications among Brazil students. In statistically significant Chi-square tests, U.S. students were more likely to have been tested for COVID-19 (68%), with 81% receiving a negative result and 19% receiving a positive result. Of the Brazil students that were tested (25%), 71% received a negative result and 29% received a positive result. Testing was reported to be more widely available within the U.S. (85% vs. 65%, respectively) as well as more likely to be free of cost within the U.S. (76% vs. 47%, respectively) (*p* = 0.012). U.S. students were more likely to agree with their city/region’s policies relating to COVID-19 (51% vs. 15%) (*p* < 0.001). Students responded to a qualitative question on their reasoning for agreeing or not. A live discussion among both groups of students gave further context to survey responses. Specifically, students in Brazil noted concern that their political leaders were not always supportive of mask-wearing and social distancing. This contributed to perceptions of inadequate risk communication and misinformation provided by leadership. While the U.S. students were more likely to agree, they still had grievances over policies and more specifically, how the government operated to carry them out. As one student stated, “…the NYC mayor and the NYS governor should have operated on one accord. Public Health officials should have been at the forefront to manage the pandemic.” Enforcement of policies and poor adherence were listed as barriers that students perceived with policies in both the U.S. and Brazil. Some of the students had issues with their regions/city policies due to the lack of enforcement of policies in place, in both countries. U.S. students also had issues with policy contradictions and the operation of policies. Brazil students reported issues with misinformation and not having enough regulation. For example, one Brazilian student reported “because they’ve recently decided to basically just open everything and to not care anymore if people are making crowds or social distancing in public spaces. There are some small measures or control policies, but it’s not enough.”

### 3.3. Risk and Protective Behaviors

Perceptions and behaviors around protective measures against COVID-19 were assessed (Table 3). The majority of U.S. (76%) and Brazil (86%) students reported no known risk factors for COVID-19 severity. Of those reported, asthma was the highest among the U.S. (13%) and being overweight/obese was the highest among Brazil (11%).

The majority of students, regardless of location, believed the most important risk factors for coronavirus contagion were crowded places, followed by people without masks, and then closed places without ventilation. Contaminated shopped items were not overwhelmingly thought of as an important risk factor for contagion. U.S. students were more likely to believe the places they were frequenting had enough sanitization procedures for them to feel safe versus the Brazil students (60.4% vs. 30.9%, respectively) (*p* = 0.004). Moreover, the majority of students believed their own sanitization procedures kept them safe from possible COVID-19 infection. Sixty percent of U.S. students and 38% of Brazil students felt they were doing enough; 28% of U.S. and 46% of Brazil students felt they were partially doing enough, and 11% of U.S. students and 16% of Brazil students did not feel as though they were doing enough.

Overall, 88% of students reported proper PPE use, irrespective of location. However, there were significant differences in the number and type of PPE used, as well as protective behavior during the pandemic. U.S. students were more likely to report using two different types of PPE (59%), the majority consisting of washable and/or disposable masks. Brazil students were most likely to use only one type (64%), that being washable masks. U.S. students were less likely to be staying at home and leaving their house only when necessary, versus the Brazil students (30% vs. 62%, respectively), more likely to leave their house by choice despite the pandemic (11% vs. 2%, respectively), and more likely to leave their house for work (34% vs. 9%, respectively) (*p* = 0.001).

### 3.4. Social World and Mental Health

Perceptions about the home social environment, relationships with family and friends, and resulting mental health impacts were reported (Table 4). All students, regardless of location, were not attending in-person classes. Additionally, all students reported living in a house or apartment, with the majority of students (81%) reported having reliable internet. Those who reported they felt well about their home environment were more likely to report they felt well about how they were handling the pandemic (*p* < 0.001), with no statistically significant differences between students in the U.S. versus Brazil.

For social relations during the pandemic, 64% of U.S. students and 51% of Brazil students stated they were not having difficulty maintaining social relationships. Out of those that did report having difficulties, lack of time was the most common reason among U.S. students (25%), and lack of volition to socialize due to mental health alterations complicating the maintenance of social relationships was most common among Brazil students (31%).

For those who sought treatment related to mental health relating to a particular aspect of the pandemic, the most pronounced reason was monetary difficulties. Other reasons included grief from the passing of loved ones and/or the added stress from caregiving of children or family members. There were no significant differences between countries on if employment changes during the pandemic impacted one’s mental health with most reporting no negative mental health impact.

## 4. Discussion

Our study population consisted of healthcare students with advanced degree programs and thus were well informed on pandemic issues and prevention knowledge. Through their self-reported results, we were able to see the influence of society and culture that each country experiences. While our study population is not exhaustive of the country’s demographics, it does give insight into the student experience during a pandemic and some of the issues that many have faced during this time.

Students’ responses on whether they agreed with their city and/or region’s policies around COVID-19 differed greatly between countries as the U.S. students were more likely to have confidence in political leadership. This follows suit with the messaging coming from both medical and political leaders did not always correspond and thus led to confusion among the public, particularly when it came to mask-wearing. This lack of knowledge of public health principles and the overall politicization of COVID-19 regarding knowledge, quarantine, and tracing efforts have demonstrated that “it is important to ensure that properly trained personnel are empowered to act in a purely nonpolitical manner” [14].

Messaging around prevention procedures from political leaders, healthcare and public health leaders, in addition to social media may have influenced the students’ responses as U.S. students were more likely to use disposable and washable masks as well as more likely to use Lysol-type sprays as a sanitization method. Google search trends were analyzed and across ten keywords the terms “face mask”, “Lysol”, and “COVID stimulus check” were the most frequent search terms in the U.S. in that order, respectively [15]. This validates the self-reported usage of Lysol-type sprays within the U.S. students sampled., In contrast, students from Brazil were more likely to use washable masks and few reported using sprays to sanitize.

Employment was another point of divergence between students across the U.S. and Brazil as approximately half of the students from the U.S. were older and employed compared with the Brazilian student population. This outcome may be due to the majority of Brazilian students being undergraduate compared with a greater number of graduate students from the U.S. involved in work outside of the classroom. Our U.S. participants being in graduate-level healthcare programs, as well as a decent portion engaged in the frontline workforce, even while in school, could have led to a higher level of awareness and understanding of disease transmission. Essential workers including healthcare workers, grocery store staff and transit operators are more likely to be African American, which relates to our U.S. student population being prominently Black or African American and involved in healthcare or essential work fields [16]. In a separate study assessing Brazilians’ COVID-19 knowledge around symptoms, transmission, and prevention study authors found that overall participants had a decent level of basic information. However, those that were female, of younger age, had a higher education level and were in economically and socially superior areas had better basic knowledge [17].

In our study population, U.S. students were more likely to have been tested for COVID-19 compared to Brazilian students. This could be tied into availability as it was also reported that tests were more widely available and free among U.S. students. This may also relate to the vaccination rates in these, as 48.2% of the U.S. population is fully vaccinated compared to only 14.0% of the Brazilian population (at the time of this reporting) [18]. Even with testing for COVID-19, disparities exist as those with limited monetary funds may not have access to a car or public transit to get to a testing site [16]. In New York City, racial composition and socioeconomic status (SES) were significant indicators of whether or not a person was tested as well as if their test was positive. Based on ZIP codes (five digit postal code), neighborhoods containing nonwhite residents and lower socioeconomic status (SES) scores were more likely to test positive for COVID-19 [19]. While most of our study population did not report severe COVID-19 disease risk factors, the most frequently reported pre-existing conditions included asthma and obesity.

In our study population, participants who had never had counseling before sought out mental health treatment during the pandemic, in addition to participants that were already regularly seeking mental health treatment before the pandemic. Our findings are substantiated by various studies on student experience during the pandemic, revealing a variety of self-reported psychological impacts and experiences. A web-based survey assessing students’ perceptions across seven different universities found that 59% (*n* = 2534) of the participants reported high levels of psychological distress [1]. A second study of university students demonstrated that mental health status was influential on risk and protective behaviors. Behavioral determinants included thoughts around coping with self-isolation and avoidance of loneliness [2]. In an additional study, protective factors against psychosocial impacts included being non-Hispanic White, being of a higher SES, and being able to spend at least two hours per day outside, demonstrating the influence of differential risk factors across social determinants [1]. In a study measuring SES by perceived social class, students of lower SES were more likely to experience psychological impacts of the pandemic [3]. These important findings warrant further study.

Additionally, our study population consisted of students engaged in distance online courses during the time of the survey. This has implications for changes in the social aspects of learning. With the increasing use of learning platforms such as Zoom, students are “dedicating cognitive resources” impacting their cognitive load leading to “Zoom fatigue” [20]. In prior studies, while some students adapted well to online learning platforms, others struggled, particularly for those without previously established social support [2]. This further endorses our findings and validates the need for administrators to be aware of the issues and challenges students may face, especially during times of change that have been brought about by the COVID-19 pandemic, thus highlighting the need for social and emotional support for students.

Based on the Abraham Maslow hierarchy, one must meet one’s basic physiological, safety, and love and belonging needs before achieving higher functions such as academic success. COVID-19 can cause tension in these needs of university students still developing their identity. A popular slogan that “students must Maslow before they can Bloom” [3,8] demonstrates the need for proactive steps to be taken by administrations to support the needs of students mentally, emotionally, socially, economically, etc., especially in times of hardships and evidently during the COVID-19 pandemic [1]. Proactive steps should also be put in place to assist students in building resilience and recovery from lasting effects such as activities focused on health and wellness, socialization, academic success, physical and mental health, and leadership and volunteerism [3].

Some limitations of this study include the cross-sectional nature of the questionnaire, which consisted of predominantly closed-ended questions making it difficult to explore the answers in greater depth and infer causality [21]. The results also reflect the perception of students in the period of collection of responses and are subject to changes in perceptions according to the evolution of knowledge about the disease. As responses were self-reported, they may be subject to participant recall bias [22], introspective ability and interpretation of questions. In addition, there may have been difficulty in comprehending cultural subtleties across languages, as the questionnaire sought to cover both countries. Another highlight is that the group of students who participated in the study are from the health field and were familiar with the topic of study. Therefore, the results obtained cannot be expanded or generalized to students from other areas of knowledge.

Our study findings illustrate the cultural and social differences that students may experience based on their country of residence. Risk and protective behaviors as well as lifestyles are influenced by politics, culture, societal expectations, and messaging. Understanding these relationships can further improve our ability to face global public health crises. Our study has also demonstrated the need for university preparedness in order to support students’ diverse needs. Recognition of these differences along with awareness of students’ support needs such as food security, mental health services, and social outlets can help build resilience and improve educational outcomes in times of crisis.

## 5. Conclusions

Our research population was undergraduate and graduate healthcare students from two universities from the U.S. and Brazil. Some of our findings align with research findings from the general population. Our sample population (educated healthcare students) was well informed on pandemic issues and prevention knowledge. On the other hand, our findings highlight the need for awareness in social and cultural issues as well as in students’ material needs. The COVID-19 pandemic has resulted in aggravated psychological and family issues. Due to stay-at-home orders, students had their schooling conditions hampered, in addition to facing effects of social isolation. Students from both countries have experienced these issues differently, influenced by the particularities of their backgrounds. Both groups of students emphasized the importance of political influences and communication on risk perceptions. A coherent message from the government is essential to guide citizens in following prevention measures in facing health crises. Additionally, incorporating cultural content into solutions can help address the social and cultural differences in risk communication and prevention in pursuit of better outcomes globally.

The COVID-19 pandemic has also revealed new requirements for higher education. Our study pointed out some important issues to support students during times of crisis aiming to improve student outcomes. Thus, university preparedness should consist of training to recognize these issues and the development of protocols to address shortcomings. Overall, we hope that our study results will inform future educational efforts in intercultural learning to address major public health crises.

## Figures and Tables

**Table 1:** Demographics of student population.

	United States (*n* = 53)	Brazil (*n* = 55)	*p*-Value
Age			<0.001
Mean (Range)	29 (22–56)	22 (17–31)	
Gender			0.006
Male	11 (21%)	27 (49%)	
Female	41 (77%)	28 (51%)	
Transgender Male	1 (2%)	0	
Race			
White	8 (15%)	-	
Black or African American	26 (49%)	-	
Asian	10 (19%)	-	
Other	9 (17%)	-	
Ethnicity			
Hispanic or Latino/a	45 (84%)	-	
Non-Hispanic or Latino/a	6 (11%)	-	
Do Not know	1 (2%)	-	
Prefer not to answer	1 (2%)	-	
Born Outside the U.S.			<0.001
No	34 (64%)	0	
Yes	19 (36%)	55 (100%)	
Undergraduate/Graduate Course			<0.001
Public Health	45 (85%)	0	
Medicine	5 (9%)	51 (93%)	
MD/MPH	1 (2%)	0	
Psychology	2 (4%)	4 (7%)	
Health Professional			<0.001
No	27 (51%)	47 (86%)	
Yes	26 (49%)	8 (15%)	
Employed Before the Pandemic			<0.001
No	9 (17%)	49 (91%)	
Yes	44 (83%)	5 (9%)	
Current Employment Status			<0.001
Employed	35 (67%)	6 (11%)	
Unemployed	17 (33%)	48 (89%)	
Employment Satisfaction			0.101
Satisfied with Job	16 (47%)	5 (83%)	
Not Satisfied with Job	18 (53%)	1 (17%)	
Employment Seeking			<0.001
Not Seeking Employment	6 (35%)	45 (94%)	
Seeking Employment	11 (65%)	3 (6%)	

**Table 2:** Student perceptions and experiences around COVID-19 physical characteristics and symptomatology.

	United States (*n* = 53)	Brazil (*n* = 55)	*p*-Value
COVID-19 # of Symptoms^1^			0.840
0	23 (46%)	19 (39%)	
1–2	12 (24%)	15 (31%)	
3–4	9 (18%)	8 (16%)	
5–8	6 (12%)	7 (14%)	
COVID-19 Symptoms Experienced^2^			
Fatigue	17	15	0.325
Headache	13	16	0.696
Loss of Taste or Smell	11	6	0.087
Sore Throat	10	13	0.629
Dry Cough	10	12	0.819
Fever	8	10	0.764
Difficulty Breathing or Shortness of Breath	7	7	0.820
Chest Pain or Pressure	3	3	0.891
Medication Taken for COVID-19			0.324
No	50 (94%)	49 (89%)	
Yes	3 (6%)	6 (11%)	
COVID-19 Tests			<0.001
No Tests Taken	17 (32%)	41 (75%)	
Negative Result	29 (55%)	10 (18%)	
Positive Result	7 (13%)	4 (7%)	
COVID-19 Test Availability			0.012
Not Widely Available	4 (8%)	15 (27%)	
Widely Available and Free of Cost	40 (76%)	26 (47%)	
Widely Available, but Not Free of Cost	5 (9%)	10 (18%)	
Do Not Know	4 (8%)	4 (7%)	
Agree with City/Region COVID-19 Policies			<0.001
No	3 (6%)	12 (22%)	
Yes	27 (51%)	8 (15%)	
Partially	19 (36%)	27 (49%)	
Does Not Apply	4 (8%)	8 (15%)	

^1^ Number of symptoms totaled for each student and categorically grouped. ^2^ Distinct variables answering yes/no of symptoms experienced.

**Table 3:** Student perceptions and experiences around risk and protective behaviors during the COVID-19 pandemic.

	United States (*n* = 53)	Brazil (*n* = 55)	*p*-Value
Personal Risk Factors			0.130
None	40 (76%)	47 (86%)	
Asthma	7 (13%)	1 (2%)	
High Blood Pressure/Heart Disease	1 (2%)	0	
Overweight/Obesity	5 (9%)	6 (11%)	
Smoking	0	1 (2%)	
Most Important Risk Factor Opinion ^1^			
Crowded Places	47	55	0.270
People Without Masks	42	40	0.428
Closed Places Without Ventilation	38	35	0.371
Contaminated Shopped Items	12	14	0.732
Enough Sanitization at Places Frequented			0.004
No	6 (11%)	17 (31%)	
Yes	32 (60%)	17 (31%)	
Partially	15 (28%)	21 (38%)	
Enough Self-Sanitization			0.069
No	6 (11%)	9 (16%)	
Yes	32 (60%)	21 (38%)	
Partially	15 (28%)	25 (46%)	
Number of Sanitization Procedures Implemented			0.249
1	14 (26%)	13 (24%)	
2–3	20 (38%)	29 (53%)	
4–5	19 (36%)	13 (24%)	
Sanitization Procedures Implemented ^1^			
Alcohol 70% Gel	31	41	0.077
Alcohol 70% Spray	30	40	0.079
Soap	40	40	0.745
Household Bleach	21	14	0.116
Lysol-Type Sprays	29	6	<0.001
Properly Using PPE			0.121
No	0	1	
Yes	50	45	
Partially	3	9	
Number of PPE Used			0.002
1	15 (28%)	35 (64%)	
2	31 (59%)	18 (33%)	
3	6 (11%)	1 (2%)	
4	1 (2%)	1 (2%)	
PPE Used ^1^			
Washable Mask	40	52	0.005
Disposable Mask	48	22	<0.001
Face Shield	8	3	0.098
Disposable Cover	3	1	0.291
Pandemic Behavior			0.001
Staying at Home. All Needs Come from Delivery Services	1 (2%)	2 (4%)	
Staying at Home. Leave Only When Necessary	16 (30%)	34 (62%)	
Usually at Home. Leave to Meet Friends Sometimes	12 (23%)	13 (24%)	
Frequently Leave House by Choice Despite Pandemic	6 (11%)	1 (2%)	
Frequently Leave House for Work	18 (34%)	5 (9%)	

^1^ Distinct variables answering yes/no of use.

**Table 4:** Student perceptions and experiences around social world and mental health during the COVID-19 pandemic.

	United States (*n* = 53)	Brazil (*n* = 55)	*p*-Value
Living Location			
House/Apartment	53 (100%)	55 (100%)	
Household			0.308
Self	3 (6%)	1 (2%)	
Parents/Family	42 (79%)	50 (91%)	
Spouse/Partner	4 (8%)	1 (2%)	
Friends	4 (8%)	3 (6%)	
Attending In-Person Class			
No	53 (100%)	54 (100%)	
Yes	0	0	
Reliable Internet			0.556
Always	43 (81%)	44 (80%)	
Sometimes	9 (17%)	11 (20%)	
Rarely	1 (2%)	0	
Feeling Well about Home Environment			0.998
No	2 (4%)	2 (4%)	F
Yes	33 (62%)	34 (62%)	
Partially	18 (34%)	19 (35%)	
Social Relationships			0.698
Not Contacted Friends and Family	5 (9%)	3 (6%)	
Contacted Friends and Family Just A Few Times	22 (42%)	28 (51%)	
Contacted Friends and Family A Lot	9 (17%)	7 (13%)	
Contacted Friends and Family Regularly	17 (32%)	17 (31%)	
Social Relationship Difficulty^1^			
No Difficulty	34	38	0.164
Lack of Time	13	11	0.571
Lack of Availability	11	6	0.160
Lack of Volition	8	17	0.051
Lack of Reliable Internet/Technology	2	3	0.678
Feeling Around the Pandemic			0.793
Doing Well	15 (28%)	18 (33%)	
Living One Day at a Time	24 (45%)	23 (42%)	
Overwhelmed with Problems	3 (6%)	5 (9%)	
Going Through Difficulties	4 (8%)	5 (9%)	
Really Tough	7 (13%)	4 (7%)	
Mental Health			0.624
Never Had Counselling	30 (57%)	27 (49%)	
Regular Counselling and Have Continued	4 (8%)	5 (9%)	
Had Counselling My Lifetime	13 (25%)	19 (35%)	
Sought Counseling Since the Pandemic Started	6 (11%)	4 (7%)	
Employment Changes Impact on Mental Health			0.275
No Impact on Mental Health	20 (41%)	14 (58%)	
Impacted Mental Health	13 (27%)	6 (25%)	
Partially Impacted Mental Health	16 (33%)	4 (17%)	

^1^ Distinct variables answering yes/no of use.

## Data Availability

The data presented in this study are available on request from the corresponding author.
